# Expression of the *Aeluropus littoralis AlSAP* Gene Enhances Rice Yield under Field Drought at the Reproductive Stage

**DOI:** 10.3389/fpls.2017.00994

**Published:** 2017-06-12

**Authors:** Thaura Ghneim-Herrera, Michael G. Selvaraj, Donaldo Meynard, Denis Fabre, Alexandra Peña, Walid Ben Romdhane, Rania Ben Saad, Satoshi Ogawa, Maria C. Rebolledo, Manabu Ishitani, Joe Tohme, Abdullah Al-Doss, Emmanuel Guiderdoni, Afif Hassairi

**Affiliations:** ^1^Departamento de Ciencias Biológicas, Universidad IcesiCali, Colombia; ^2^International Center for Tropical AgricultureCali, Colombia; ^3^UMR Amélioration Génétique et Adaptation des Plantes Méditerranéennes et Tropicales, Centre de Coopération Internationale en Recherche Agronomique pour le DéveloppementMontpellier, France; ^4^Department of Plant Production, College of Food and Agricultural Sciences, King Saud UniversityRiyadh, Saudi Arabia; ^5^Biotechnology and Plant Improvement Laboratory, Centre of Biotechnology of Sfax, University of SfaxSfax, Tunisia; ^6^Graduate School of Agricultural and Life Science, Department of Global Agricultural Science, The University of TokyoTokyo, Japan; ^7^Centre of Biotechnology of SfaxSfax, Tunisia

**Keywords:** *AlSAP*, A20/AN1 stress-associated protein, drought, reproductive stage, rice, yield

## Abstract

We evaluated the yields of *Oryza sativa* L. ‘Nipponbare’ rice lines expressing a gene encoding an A20/AN1 domain stress-associated protein, AlSAP, from the halophyte grass *Aeluropus littoralis* under the control of different promoters. Three independent field trials were conducted, with drought imposed at the reproductive stage. In all trials, the two transgenic lines, RN5 and RN6, consistently out-performed non-transgenic (NT) and wild-type (WT) controls, providing 50–90% increases in grain yield (GY). Enhancement of tillering and panicle fertility contributed to this improved GY under drought. In contrast with physiological records collected during previous greenhouse dry-down experiments, where drought was imposed at the early tillering stage, we did not observe significant differences in photosynthetic parameters, leaf water potential, or accumulation of antioxidants in flag leaves of AlSAP-lines subjected to drought at flowering. However, AlSAP expression alleviated leaf rolling and leaf drying induced by drought, resulting in increased accumulation of green biomass. Therefore, the observed enhanced performance of the AlSAP-lines subjected to drought at the reproductive stage can be tentatively ascribed to a primed status of the transgenic plants, resulting from a higher accumulation of biomass during vegetative growth, allowing reserve remobilization and maintenance of productive tillering and grain filling. Under irrigated conditions, the overall performance of AlSAP-lines was comparable with, or even significantly better than, the NT and WT controls. Thus, AlSAP expression inflicted no penalty on rice yields under optimal growth conditions. Our results support the use of AlSAP transgenics to reduce rice GY losses under drought conditions.

## Introduction

Field drought could severely alter global rice production (740 million tons from over 160 million hectares of paddy in 2013^1^). Twenty percent of Asian rice acreage is drought-prone, and the frequency, extent, and intensity of drought events are amplified by increasing climate instability (Pandey and Bhandari, 2007). Drought mainly occurs in rain-fed and upland rice growing systems; however, the need to save irrigation water in lowland paddy fields may also lead to periods of drought in more favorable environments. Rice is sensitive to drought over its growth cycle; however, drought stress is most detrimental to grain yield (GY) when it occurs at the reproductive stage (from panicle initiation to fertilization). Drought limits transpiration, which is required for panicle cooling and causes spikelet sterility, while impairing proper reserve remobilization and grain filling (Prasad et al., 2008).

Grain yield under drought occuring at flowering stage is the most reliable trait for discrimination of tolerant progeny in breeding programs (Atlin, 2003; Kumar et al., 2008). Long-term efforts to identify quantitative trait loci (QTL) associated with drought-avoidance and -tolerance have identified genomic regions involved in these complex traits (Lanceras et al., 2004; Bernier et al., 2009; Courtois et al., 2009; Khowaja et al., 2009). More recently, genes influencing drought-related traits have been cloned (Uga et al., 2013; Raorane et al., 2015). Undoubtably, the re-sequencing of genetic resources [The 3,000 genomes project (Li et al., 2014)] and target population of environments-phenotyping^2^ of a broad range of accessions will accelerate the identification of new loci contributing to drought-tolerance in the near-future.

An alternative strategy is the engineering of drought-tolerance using transgenic approaches, which can use various expression systems (constitutive, tissue-specific, or drought-inducible) (Gaudin et al., 2013). While an extensive range of genes from different origins has already been introduced into rice and proven to induce some level of drought-tolerance in laboratory/greenhouse experiments, reports of challenges of transgenic rice with field-drought at the reproductive stage are limited (Gaudin et al., 2013). Transgenic drought-tolerance has primarily been achieved using genes encoding transcription factors (TFs), such as members of the AP2/ERF (Oh et al., 2009; Ambavaram et al., 2014); NAC, NAM, ATAF, and CUC (Hu et al., 2006; Jeong et al., 2010; Redillas et al., 2012); and homeodomain-leucine zipper (Liu et al., 2013) families. Genes involved in abscisic acid (ABA) (Xiao et al., 2009) and proline (You et al., 2012) synthesis, along with those encoding a late embryogenesis abundant protein (Xiao et al., 2007) and a proteinase inhibitor (Huang et al., 2007), have also improved yield under drought in field experiments. In these reports, drought was imposed at the flowering stage, and observed significant gains in yield were explained by reduced sterility, enhanced root systems, and increased tiller/panicle/grain number/size. More importantly, in these examples, no yield penalties were observed under unstressed conditions; indeed, some instances of enhanced yield in the absence of stress were reported (Ambavaram et al., 2014).

Over the last 10 years, there has been increasing interest in a class of zinc-finger proteins, the A20/AN1 domain stress-associated protein (SAP) family. These molecules exhibit structural and functional conservation among plant species (Vij and Tyagi, 2008; Giri et al., 2013). The rice genome harbors 18 *SAP* genes encoding the A20/AN1 domain, while *Arabidopsis* contains 14. Interestingly, the majority of the published attempts to constitutively express SAPs in the model plants, tobacco, *Arabidopsis*, and rice, have resulted in enhanced tolerance to multiple abiotic stress factors, including drought, salinity, cold, heat, oxidative stress, and heavy metals. Similar examples of transgenic protection have also recently been reported in durum wheat (Ben Saad et al., 2011), banana (Sreedharan et al., 2012), and cotton (Hozain et al., 2012).

Stress-associated proteins can exhibit various modes of action. Recent reports of experiments involving *Arabidopsis* AtSAP5 (Kang et al., 2011, 2013; Choi et al., 2012) and rice OsSAP1/OsSAP11 (Giri et al., 2011) indicate that SAPs can recognize and bind polyubiquitin chains, may exhibit E3-ligase activity, and act through protein–protein interactions involving their A20/AN1 zinc finger domains. SAPs also have a well-established roles in the negative regulation of ABA [AtSAP5 (Kang et al., 2013), OsSAP7 (Sharma et al., 2015)], and gibberellic acid [OsSAP11/OsDOG (Liu et al., 2011)] mediated responses. Other recent evidence indicates that AtSAP12 may have a redox sensor role via cysteine residues outside of the conserved A20/AN1 domain (Stroher et al., 2009). Ectopic accumulation of SAPs can influence the expression of abiotic stress-related genes in *Arabidopsis*, rice, and cotton; however, a function for these proteins as transcription regulators through direct DNA-binding has been suggested but not demonstrated. A recent transcript profiling report in rice indicated that overexpression of *OsSAP1* influenced the transcription of 150 genes, including 43 involved in stress responses (Dansana et al., 2014).

Based on the hypothesis that halophytes could be a valuable source of adaptive genes, we isolated an A20/AN1 SAP encoding gene, *AlSAP*, from the C4 halophyte grass *Aeluropus littoralis* (Zouari et al., 2007). AlSAP protein exhibits 79% sequence similarity with rice OsSAP9, is inducible by multiple stress factors (Ben Saad et al., 2010), and, when expressed in transgenic tobacco, durum wheat (Ben Saad et al., 2011), and the japonica rice cv. Nipponbare (*Oryza sativa* L. ‘Nipponbare’) (Ben Saad et al., 2012), confers enhanced tolerance to cold, drought, salt, and oxidative stress. Under greenhouse conditions, AlSAP transgenic rice plants survived a severe 15-day dry-down experiment (DDE) imposed at the six-leaf, early tillering stage. AlSAP-lines rehydrated at the end of the stress period yielded 50% of the grain produced under unstressed conditions, whereas wild-type Nipponbare (WT-NIP) did not recover from the treatment. Physiological investigations conducted during the DDE revealed that the enhanced performance of the transgenics was likely due to the maintenance of photosynthesis and the integrity of the photosynthetic apparatus under stress (Ben Saad et al., 2012). Importantly, over-expression of AlSAP in rice did not appear to alter its performance under unstressed greenhouse growth conditions. This contrasts with the reports of OsSAP overexpression in rice, where a reduction in GY was observed under unstressed conditions (Kanneganti and Gupta, 2008; Dansana et al., 2014).

As mentioned above, transgenic protection from drought requires testing in the field at several locations and with stress imposed at the most sensitive stage, from panicle initiation to fertilization. Also, potential detrimental or beneficial effects of transgene expression require assessment under stringent yield trials. With this aim, we set up three replicate field trials in Colombia, with drought stress imposed at the flowering stage, either through a naturally occurring dry season or by placement of the field trial under a rainout shelter. Six AlSAP-lines, along with non-transgenic (NT) azygous control lines and WT-NIP were challenged at panicle initiation stage with different soil drought conditions. During these trials, we also monitored the status of plants at the physiological and biochemical levels, to investigate AlSAP function. Parallel, irrigated trials were conducted to determine whether the constitutive expression of AlSAP alters rice yields under unstressed conditions.

Data from the three drought field trials consistently indicated that the two highest-expressing AlSAP lines out-yielded the NT and WT controls under drought field conditions. Consistent and significantly higher yields were also observed among AlSAP-lines in irrigated trials. No significant physiological or biochemical differences were observed between AlSAP-lines and WT and NT controls. Our results suggest that the superior performance of AlSAP-lines under field drought was likely due to a primed status, with beneficial features, including enhanced biomass, accumulated during vegetative growth, that facilitate improved tolerance of water deficit at the flowering stage.

## Materials and Methods

### Ethical Statement

The authors declare that the transgenic experiments described herein comply with the current biosafety laws of the country in which they were performed.

### Rice Transformation

The isolation and cloning of the *AlSAP* gene from *A. littoralis* was described previously (Ben Saad et al., 2011, 2012). The AlSAP gene and protein sequences are deposited in the GenBank (DQ885218) and UniProtKB (A1YAQ3) databases. The rice genome contains 12 SAP genes encoding A20 and AN1 domains. The relationship between AlSAP and OsSAP proteins was established by Ben Saad et al. (2010); the *SAP* gene encoding the protein sharing the highest amino acid identity (79%) is *OsSAP9* (Os07g07350) which is the likely ortholog of *AlSAP* in rice (Supplementary Figure S1).

We evaluated six AlSAP-lines and their NT azygous controls in our field trials (Supplementary Figures S2, S3). RN4, RN2, and RN5 (T5 homozygous lines) expressed *AlSAP* under control of the 35S CaMV promoter and accumulated *AlSAP* transcripts at increasing levels (Ben Saad et al., 2012). RN1 and RN3 (T3 homozygous material) expressed *AlSAP* under the control of the maize Ubiquitin1 regulatory region (Christensen et al., 1992). RN6, also a T3 homozygous material, expressed AlSAP under the rice *ACT1* gene regulatory region (Mc Elroy et al., 1990). T3 homozygous RN3, RN1, and RN6 plantlets accumulated *AlSAP* transcript at moderate, medium, and high levels in leaves, respectively, at the six leaf stage under greenhouse conditions. Each of the three lines harbored a single copy of the transgene (Supplementary Figure S3).

### Experimental Field Design

In Trial 1, six homozygous *AlSAP*-transformed lines (RN1–6), together with their transformation controls, and non-transformed WT-NIP plants were evaluated in a natural upland rain-fed field from November 2012 to March 2013 at CIAT Santa Rosa station (Villavicencio, Colombia). The transgenic and WT-NIP lines were sown in three replicate 2 m × 1 m plots with a spacing of 25 cm × 10 cm and received regular fertilization. The plants experienced a 42-day rain-free period, which began at panicle initiation and extended for approximately 28 days after attainment of 50% flowering (Supplementary Figure S5).

Trials 2 and 3 were performed in the rainout shelter facilities at CIAT headquarters (Palmira, Colombia); the drought treatment was imposed at panicle initiation stage by suspending irrigation. In Trial 2 (April–July 2013), RN2, RN5, and RN6 were evaluated, together with their NT controls and WT-NIP, while in Trial 3 (June–October 2013) only RN5 and RN6 were assessed, together with their NT controls, and WT-NIP. Lines were sown in a randomized complete block design, with three replicates. Each plot had six rows of 16 plants with a spacing of 20 cm × 10 cm. WT-NIP plants were inserted after every four transgenic line plants for comparison. Drought was imposed for 22 days (Trial 2) and 19-days (Trial 3), after which the plants received full irrigation.

For both Trials 2 and 3, a duplicate set of materials were also sown under flooded conditions in another field, 5 m away from the rainout shelter, to evaluate differences in GY between transgenic and control rice under irrigated conditions.

Harvest was completed for all trials 107–114 days after sowing (DAS).

### Soil Moisture Measurement and Calculation of Soil Water Matric Potential

The progress of drought-stress was monitored by measuring changes in soil moisture at four depths, 20, 40, 60, and 80 cm, using Aquapro sensors (Reno, NV); polycarbonate access tubes were installed at regular intervals within each replicate, and readings were taken regularly during the stress period. Humidity data were transformed to soil matric potential (Ψ_soil_) values using soil water retention curves (SWRC) built for each location. Aquapro soil moisture values (%) were converted to volumetric soil water content using the equation:

$$\begin{array}{c} \;\theta_{\mathrm{vol}}=\frac{A}{2.4}+b \end{array}$$

where 𝜃_vol_ is the volumetric soil moisture content (%), A is the Aquapro measurement value (Aquapro %), and b is a constant depending on soil type (Tromp-van Meerveld and McDonnell, 2006). SWRC were obtained experimentally using the pressure plate apparatus method (Bittelli and Flury, 2009).

### Measurement of Yield and Growth Parameters

Single plant yield and yield components were measured in each plot for all trials. Yield parameters were scored for 15 plants. Unfilled and filled grains were independently counted and weighed. Grain weight was used to determine final yields at 14% moisture content. The following agronomic traits were scored as established in the Standard Evaluation System for Rice (IRRI, 2013): flowering date, plant height (cm), total aerial biomass (g), panicle length (cm), number of tillers, number of panicles, total grain weight (g), and grain fertility (%).

### Photosynthetic Performance

The rate of light-saturated photosynthesis (*A*_sat_), stomatal conductance (*G*_s_), and intercellular CO_2_ concentration (*C*_i_) of expanded flag leaves were measured from 9:00 to 12:00 h using an LI-6400 portable photosynthesis open system (Li-Cor Biosciences, Lincoln, NE, United States). Measuring conditions were as follows: photosynthetic photon flux intensity = 1500 μmol/m^2^/s, leaf temperature = 27.0°C ± 1°C, ambient CO_2_ concentration = 380 ± 10 μmol/mol, and relative humidity = 60%. The rate of CO_2_ saturated photosynthesis (*A*_max_) was measured under the conditions described above, except that the ambient CO_2_ concentration was 1800 μmol/mol.

### Leaf Rolling, Leaf Drying, and Drought Recovery Scores

Leaf rolling (LR) was characterized at regular intervals in drought-stressed plants using a scoring scale from 0 (unrolled) to 9 (tight rolling) according to the Standard Evaluation System for Rice (IRRI, 2013). Leaf drying (LD) was evaluated at the peak of stress using a scoring system from 0 (no symptoms) to 9 (dead plant). The drought recovery score (DRR) was recorded 10 days after rewatering using a scoring system from 1 (90–100% recovery) to 9 (0–19% recovery) (IRRI, 2013). All evaluations were performed at noon using whole plants.

### Leaf Water (Ψw)

On three different occasions during Trial 2 (before stress, at stress peak, and 10 days after rewatering), Ψw was evaluated (pre-dawn) in drought-stressed plants. Measurements were performed on fully expanded flag leaves, as previously described (Turner, 1981), using a pressure chamber (Soil Moisture Equipment 3005F01) with a measurement limit of -15 MPa.

### Oxidative Damage and Antioxidant Capacity in Leaf Tissues

The antioxidant capacity was determined using the ABTS^∙+^ test system, based on the method of (Du et al., 2009). The level of lipid peroxidation in leaf tissues was measured as MDA content, following a published protocol (Ueda et al., 2013).

### Monitoring of *AlSAP* Transcript Accumulation

Procedures for Southern and northern blot analyses, and RT-PCR were the same as those detailed by Ben Saad et al. (2012). Total DNA and RNA were isolated from leaf tissue of six leaf stage seedlings, and hybridizations performed using P^32^ labeled *Npt2, Hpt*, or *AlSAP* gene probes. RT-PCR analyses were performed using RNA isolated from leaves and primers specific to *AlSAP* and the housekeeping gene, *OsExp* (Caldana et al., 2007), used as an internal expression standard (Supplementary Table S1). For samples from field grown plants, inflorescences were collected from the master tiller of three independent plants per line at flowering stage, pooled and stored. RNA isolation and northern blot procedures were performed as indicated above (Ben Saad et al., 2012).

### Statistical Analyses

Data for days to flowering, height, total aerial biomass, GY, yield components, leaf MDA, and TEAC-ABTS were subjected to General Linear Model (GLM) analysis, appropriate for a randomized complete block design. Differences between mean values from WT-NIP- and *AlSAP*-transformed lines were compared using Dunnett’s test. A GLM was also used to test the effect of drought treatment (days after the last irrigation), and repetition on the soil matric potential (Ψ_soil_) measured at a depth of 40 cm in Trials 2 and 3. All statistical analyses were performed using SAS v9.2 (SAS Institute Inc., Cary, NC, United States). Data (means ± standard errors of raw data) are presented graphically using GraphPad Prism (GraphPad PRISM for Windows v.7.0, GraphPad Software Inc., La Jolla, CA, United States).

## Results

### Drought Intensity Characterization

**FIGURE 1:**
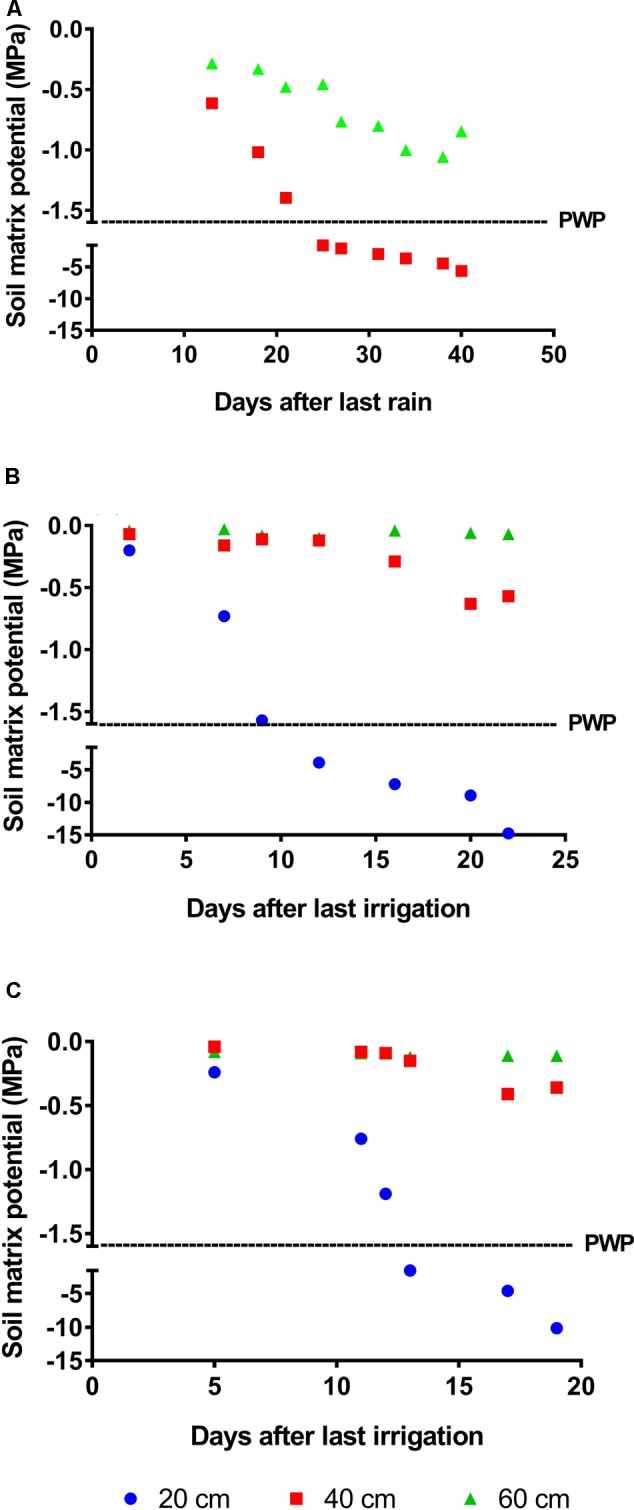
Progress of drought-stress measured as changes in ψ_soil_. **(A)** Trial 1 was performed under upland conditions in Santa Rosa (Villavicencio, Colombia). In this trial, at a depth of 40 cm the permanent wilting point (PWP, –1.6 MPa) was reached 25 days after the initiation of the rain-free period. Trials 2 and 3 were performed under the rainout facilities at CIAT headquarters (Cali, Colombia). Drought stress was imposed by suspending irrigation and allowing the soil to dry out. **(B)** In Trial 2, plants were kept under stress for 22 days, and ψ_soil_ at 40 cm reached –0.6 MPa by day 20. **(C)** For Trial 3, plants were subjected to drought for 19 days; in this trial, relatively higher ψ_soil_ at 40 cm depth (–0.45 MPa) indicated that the plants were submitted to a milder stress relative to the previous trials.

Drought intensity and duration varied among trials with harsher conditions in Trial 1 (Santa Rosa station). In this trial, drought progressed rapidly in the first 20 cm of soil, with Ψ_soil_ falling under the permanent wilting point (PWP, -1.6 MPa) 10 days after stress initiation (DASI). Changes in the 40 cm layer proceeded more slowly, with Ψ_soil_ reaching -1.6 MPa by day 22. Thereafter, Ψ_soil_ decreased steadily until the first rains occurred. A reduction in Ψ_soil_ was also observed at 60 cm depth; however, minimum values were approximately -1.0 MPa by the end of the rain-free period (**Figure 1A**). In the rainout shelter in Trial 2 (Palmira station), Ψ_soil_ in the 20 cm soil layer also fell below the PWP 10 DASI and reached very low values during the drought period (**Figure 1B**). However, Ψ_soil_ changes at depths of 40 and 60 cm were less pronounced than those in Trial 1. Drought developed in a similar fashion in Trial 3 (Palmira), although the overall stress intensity was less severe than that in Trial 2 (**Figure 1C**). No significant differences in Ψ_soil_ were detected between replicates in any trials, indicating that drought-stress progressed uniformly among replicates (Supplementary Table S2).

The Nipponbare root system normally develops in the first 40 cm of soil (Hanzawa et al., 2013). Thus, we considered changes in Ψ_soil_ at depths of 0–40 cm good indicators of the level of drought-stress experienced by plants in the field.

### Yields under Drought

In Trial 1, six homozygous *AlSAP*-transformed lines (RN1, RN2, RN3, RN4, RN5, and RN6) (generations T3–T5) were evaluated together with their azygous NT controls and WT-NIP. RN2, RN4, and RN5 harbor the *AlSAP* coding sequence under the control of the CaMV35S promoter (Ben Saad et al., 2012), while RN1, RN3, and RN6 contain one copy of a T-DNA containing the *AlSAP* coding sequence directed by the maize *Ubi-1* promoter or the rice *ACT1* promoter (Supplementary Figures S2, S3). All materials reached 50% flowering around 45–47 DAS, indicating that AlSAP accumulation did not affect plant phenology. The accumulation of AlSAP transcripts was monitored by northern blot analysis in inflorescence tissues collected from field grown plants at the initiation of drought stress imposition (Supplementary Figure S4). AlSAP transcript accumulation in these tissues was comparable in RN5 and RN6 and lower in RN2. On the other hand, the level of accumulation in RN1, RN3, and RN4 was very low compared to their respective levels in leaves of young plants at the previous generation. This was particularly surprising for the RN1 line which was identified as a medium expresser. Previous literature report describes high activity of the maize *Ubi-1* regulatory regions in reproductive organs of rice (Cornejo et al., 1993). This may tentatively be ascribed to a regulation of the maize ubiquitin promoter under field conditions or to a silencing of the transgene.

**FIGURE 2:**
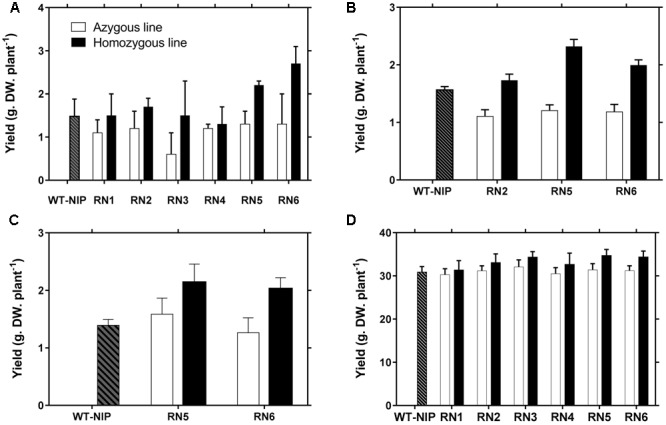
Improved grain yield in AlSAP-lines exposed to drought stress. **(A)** Grain yield (GY) of WT-NIP and AlSAP-lines RN1–6 in Santa Rosa (Trial 1). **(B,C)** As for the Santa Rosa trial, RN5 and RN6 outperformed WT-NIP and their NT controls under the drought conditions imposed in Trials 2 and 3. **(D)** Higher GY for AlSAP-lines RN1–6 was also observed in the irrigated control conducted in Trial 2.

In this trial, the plants were subjected to 42 days of drought stress from panicle initiation to 28 days after the plants reached 50% flowering, which caused a marked reduction in Ψ_soil_. Under these severe stress conditions, RN2, RN5, and RN6 were able to maintain their green biomass (Supplementary Figure S6) and exhibited higher GY than WT-NIP and NT controls, with relative gains of 46, 97, and 115%, respectively, compared with NT controls (**Figure 2A**). The NT controls tended to be more affected by drought stress than WT-NIP and, although this difference was not significant, it was consistent. However, the RN1 and RN3 NT control lines exhibited significantly lower GY (*p* < 0.001) than either the WT-NIP or NT controls of the other transformation events following the field drought-stress. This depressive effect could be due to somaclonal variation. We also see previously that these two lines eventually expressed AlSAP at a low level under field conditions. Interestingly, a beneficial effect of *AlSAP* expression was still observed in these variants; however, this was not sufficient to cause yields significantly higher than those of WT-NIP. The four other lines could be grouped into two classes according to their performance relative to WT-NIP, and their level of accumulation of *AlSAP* transcripts. The low level of transcript expression in the RN4 line did not result in enhanced yield protection under drought stress at flowering, whereas the medium and high expression levels found in RN2, RN5, and RN6 conferred enhanced tolerance to these lines. Therefore, lines RN1, RN3, and RN4 were not included in subsequent drought trials.

Trials 2 and 3 were conducted using a rainout shelter to impose drought-stress. In Trial 2, RN2, RN5, and RN6 consistently achieved higher GYs than their NT controls, exhibiting GY increases of 57, 92, and 69%, respectively (**Figure 2B**). RN5 and RN6 also exhibited superior GY in Trial 3 (**Figure 2C**). The higher GYs were associated with significantly higher numbers of panicles (*p* < 0.001), higher fertility (*p* < 0.001), and higher accumulation of biomass in AlSAP-lines (*p* < 0.001) (**Table 1**). All lines flowered around the same date, 53–55 DAS; thus, they were at very similar physiological stages when subjected to drought (**Table 1**).

**Table 1:** Days to flowering, height, total aerial biomass, grain yield, and yield components under irrigated and drought stress conditions in the rainout shelter (Trial 2).

Lines	Days to Flowering	Height (cm)	Total biomass (g DW)	Panicle number per plant	Panicle Length (cm)	Grain Yield per plant	Grain number per spikelet	Fertility
**Irrigated (control)**								
WT-NIP	48.33 ± 0.58	78.44 ± 0.88	33.45 ± 0.44	18.00 ± 1.00	18.17 ± 0.79	30.89 ± 1.28		
RN2	46.30 ± 2.08	79.78 ± 1.30	32.38 ± 1.76	18.67 ± 1.22	18.33 ± 0.37	33.10 ± 1.97ˆc		
RN5	47.00 ± 1.00	78.56 ± 1.01	36.46 ± 0.40ˆc	19.89 ± 1.27ˆc	20.11 ± 0.60ˆc	34.72 ± 1.40ˆc		
RN6	47.66 ± 0.58	76.56 ± 2.01ˆc	37.20 ± 0.58ˆc	20.67 ± 2.12ˆc	19.33 ± 1.12ˆa	34.38 ± 1.35ˆc		
**Drought**								
WT-NIP	55.00 ± 0.10	55.97 ± 0.54	6.06 ± 0.99	19.23 ± 0.21	14.92 ± 0.15	1.40 ± 0.41	180.24 ± 13.25	9.53 ± 1.49
RN2	55.00 ± 0.80	57.83 ± 3.47	6.21 ± 1.17	19.80 ± 1.85	14.23 ± 1.51	1.73 ± 0.48	174.33 ± 11.90	8.05 ± 1.23ˆa
RN5	53.67 ± 0.48ˆc	52.00 ± 4.04ˆc	9.60 ± 1.69ˆc	22.61 ± 1.91ˆc	15.48 ± 1.40	2.32 ± 0.56ˆc	176.89 ± 11.40	15.37 ± 2.00ˆc
RN6	53.00 ± 0.84ˆc	53.28 ± 3.16ˆb	9.71 ± 1.95ˆc	22.44 ± 3.13ˆc	15.38 ± 0.89	1.99 ± 0.41ˆc	178.22 ± 9.18	15.63 ± 1.83ˆc

*Values are means ± SD (*n* = 18). ^a^*P* < 0.05; ^b^*P* < 0.01; ^c^*P* < 0.001.*

In Trial 2, lines RN1–6 were also grown under irrigated conditions, along with their NT controls and WT-NIP to evaluate the effect of AlSAP accumulation in the absence of drought-stress. Interestingly, under irrigated conditions, all NT controls behaved in the same manner as WT-NIP, while the transgenic lines tended to outperform both types of controls (**Figure 2D**). This indicates that the depressed background represented by the RN1 and RN3 NT controls influence performance only under drought conditions and, could, therefore, affect stress-related traits. Irrigated plots were also established in the third field trial. Under irrigation, the accumulation of AlSAP significantly enhanced GY (*p* < 0.001). As observed under drought-stress, this effect was related to both higher numbers of panicles (*p* < 0.001) and accumulation of vegetative biomass (*p* < 0.001) (**Table 1**).

Together, these results demonstrate that *AlSAP* expression increases GY under drought-stress imposed at the reproductive stage, through a mechanism that involves the maintenance of vegetative biomass, a higher number of panicles, and enhanced grain fertility. Furthermore, AlSAP-lines also exhibited higher yields, related to higher panicle numbers and increased vegetative biomass, when grown under irrigation.

### Sensitivity to Drought

**FIGURE 3:**
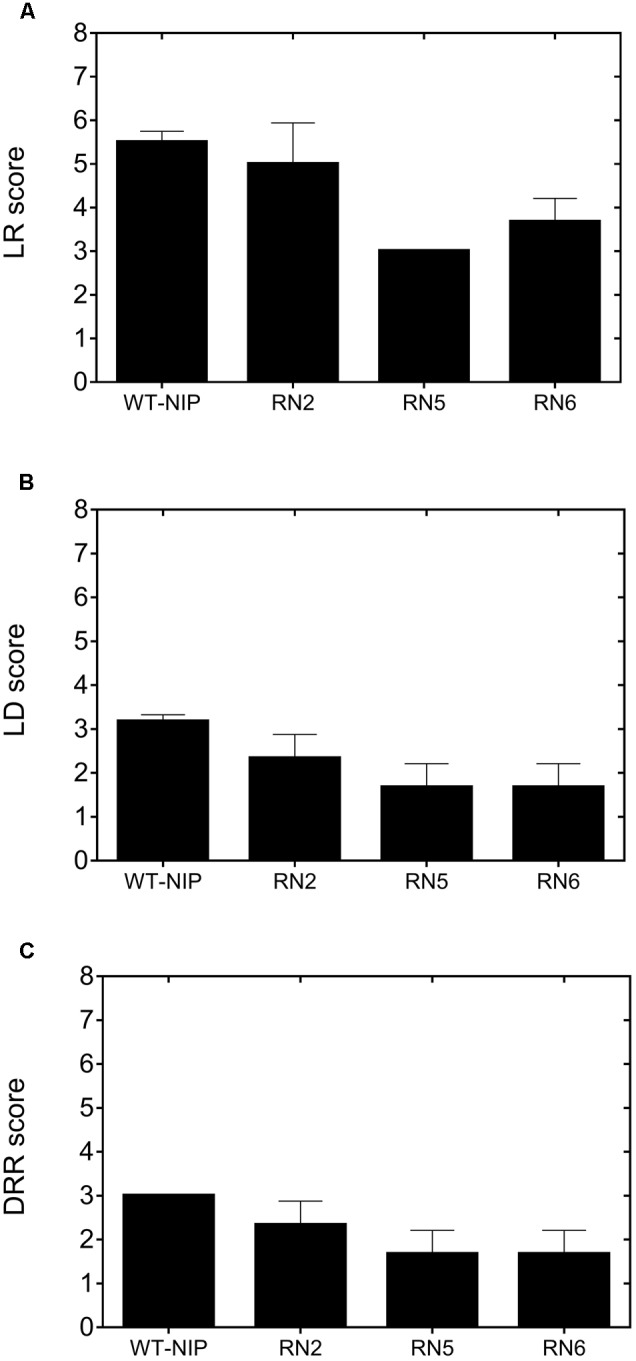
Leaf rolling (**A**, LR), leaf drying (**B**, LD), and drought recovery scores (**C**, DRR) exhibited by AlSAP-lines and WT-NIP. AlSAP-lines were less sensitive to drought and exhibited better recovery after rewatering. LR and LD were recorded at the peak of stress, while DRR was evaluated 10 days after rewatering. All measurements were taken at noon.

In Trial 2, we compared the drought sensitivity of AlSAP-lines, their NT controls, and WT-NIP by monitoring the progress of LR, LD, and DRRs (IRRI, 2013). LR appeared earlier and proceeded more rapidly in WT-NIP plants, reaching a score of 5.5 at the peak of stress (22 DASI) (**Figure 3A**), indicating that leaves were fully cupped. At this time, LR scores for RN5 and RN6 were 3.0 and 3.6 (leaves folding), respectively. At peak stress, the LD score for WT-NIP was 3.2 (drying up to ¼; of the length of the leaf), while RN5 and RN6 scores were 1.6 (slight tip drying) (**Figure 3B**). DRR was evaluated 10 days after rewatering, RN5 and RN6 showed better recovery (DRR = 1.7) than WT-NIP (DRR = 3.0). These results indicate that the best-performing AlSAP lines were much less sensitive to drought and exhibited improved recovery after rehydration compared with controls (**Figure 3C**).

### Leaf Water Potential (ψ_w_)

In rice, the ability to maintain ψ_w_ at the flowering and grain filling stages is related to variation in GY under drought-stress (Sibounheuang et al., 2006). Furthermore, there is a negative correlation between ψ_w_ and the percentage of spikelet sterility in rice plants subjected to drought at flowering (Jongdee et al., 2002). Therefore, in Trial 3 we evaluated whether the GY differences observed among RN5, RN6, and WT-NIP were related to an improved ability to maintain higher ψ_w_.

**FIGURE 4:**
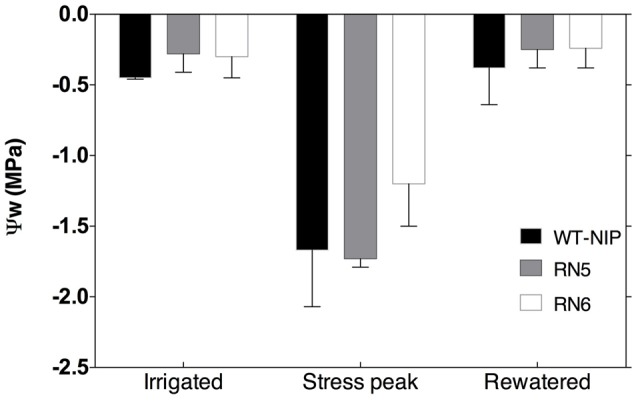
Pre-dawn leaf water potential (ψ_w_) in RN5, RN6, and WT–NIP, measured before stress (irrigated), at peak stress, and 10 days after rewatering.

No significant differences in ψ_w_ were detected before the imposition of drought stress, with ψ_w_ values oscillating between -0.15 and -0.50 MPa (**Figure 4**). After 19 days under drought-stress, ψ_w_ decreased significantly in all lines, reaching values of -0.90 to -2.10 MPa. RN6 plants showed slightly, but not significantly, higher ψ_w_ than RN5 and WT-NIP. Rewatering of plants resulted in ψ_w_ recovery to pre-drought values, with no significant differences among lines (**Figure 4**).

### Photosynthetic Performance

**FIGURE 5:**
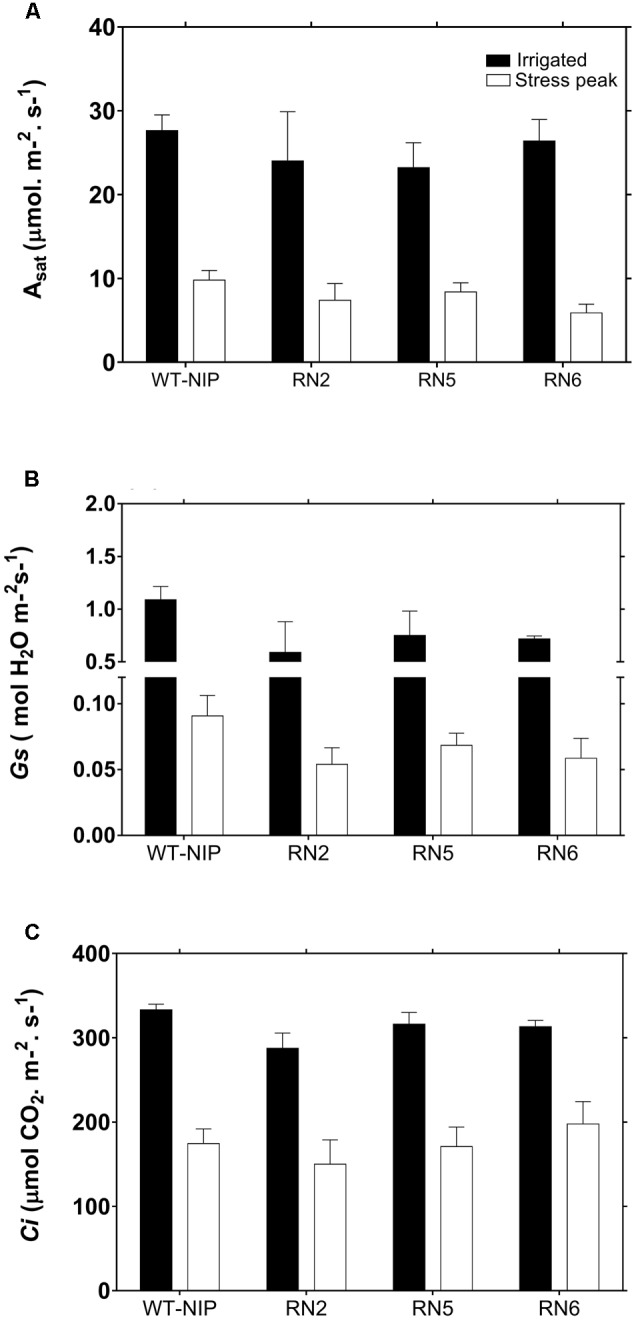
Instantaneous photosynthesis rate (**A**, *A*_sat_), stomatal conductance (**B**, *G*_s_), and internal CO_2_ concentration (**C**, *C*_i_) for WT-NIP and AlSAP-lines under irrigated and drought conditions. No significant differences were observed between AlSAP-lines and WT-NIP under the stress conditions imposed in Trial 2. Photosynthetic performance was evaluated before (irrigated) and 19 days after drought-stress was imposed (stress peak). [CO_2_]_ambient_ = 380 mmol/mol, photosynthetic photon flux intensity = 1500 mmol/m^2^/s, RH = 60%, Leaf_Temp_ = 28°C.

Flag leaves of AlSAP-lines and WT-NIP showed a marked reduction in *A*_sat_ under the stress conditions imposed in Trial 2 (**Figure 5A**). Plants exhibited *A*_sat_ rates of approximately 30 μmol/m^2^/s before the imposition of drought; however, rates were reduced by approximately 70% at the peak of drought stress. The reduction in *A*_sat_ was matched by a strong diminution in *G*_s_ and *C*_i_ (**Figures 5B,C**), indicating that stomatal closure intensely limited photosynthesis.

**FIGURE 6:**
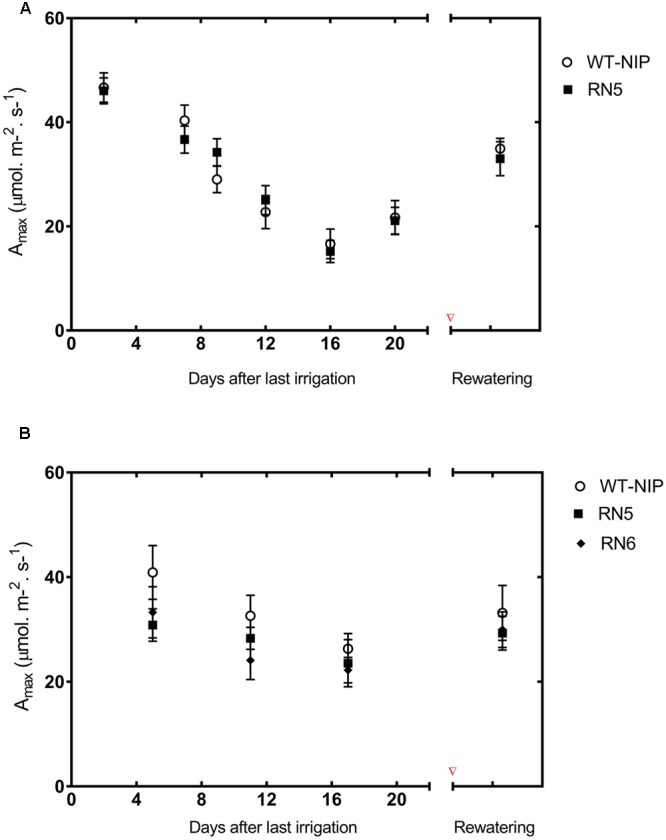
Drought-induced changes in CO_2_ saturated photosynthesis rate (*A*_max_) during Trials 2 **(A)** and 3 **(B)**. Regular measurements were taken during the drought stress period. Experimental plots were re-irrigated when leaves started to fold, and *A*_max_ was measured again 10 days later. [CO_2_]_ambient_ = 1800 mmol/mol, photosynthetic photon flux intensity = 1500 mmol/m^2^/s, RH = 60%, Leaf_Temp_ = 28°C

To establish whether the reduction in *A*_sat_ was exclusively due to diffusive limitations, we evaluated the photosynthetic responses of RN5 and WT-NIP under a saturating CO_2_ concentration (*A*_max_) at regular intervals during the drought period and 10 days after rewatering (**Figure 6A**). Under irrigated conditions, *A*_max_ was approximately 46 μmol/m^2^/s; however, it reduced progressively during drought reaching values of 21 μmol/m^2^/s, at the peak of stress. After rewatering, *A*_max_ did not fully recover to pre-drought levels. These results indicate that the reduction of photosynthesis also involved biochemical damage. The similar responses exhibited by RN5 and WT-NIP suggest that *AlSAP* expression did not offer protection to the photosynthetic machinery under the high stress levels imposed in Trial 2.

We also evaluated *A*_max_ under the milder stress conditions imposed in Trial 3. RN6 was also included in this evaluation. Similar to Trial 2, we observed a progressive diminution of *A*_max_ in all lines as drought-stress intensified (**Figure 6B**). However, *A*_max_ was reduced to a lesser extent in AlSAP-lines, and recovered almost entirely after rehydration. At the peak of stress, *A*_max_ in AlSAP-lines was reduced by 24% compared to the initial rate, while in its NT control *A*_max_ diminished by 52%. After reirrigation, AlSAP-lines showed a 95% recovery of *A*_max_; however, the NT control attained only 59%. Thus, it appears that *AlSAP* may exert a “protective” effect on photosynthesis, specifically at the biochemical level, and that this effect is modulated by the intensity of drought-stress.

### Oxidative Stress

Previous work on *AlSAP*-transformed rice demonstrated that detached leaves of AlSAP-lines maintained high chlorophyll content than controls after exposure to high concentrations of H_2_O_2_, indicating tolerance to oxidative stress. Additionally, AlSAP-lines showed enhanced expression of the genes *OsSodA* and *OsAPX1*, encoding the enzymes manganese superoxide dismutase and ascorbate peroxidase, respectively, which are involved in oxidative protection (Ben Saad et al., 2012).

**FIGURE 7:**
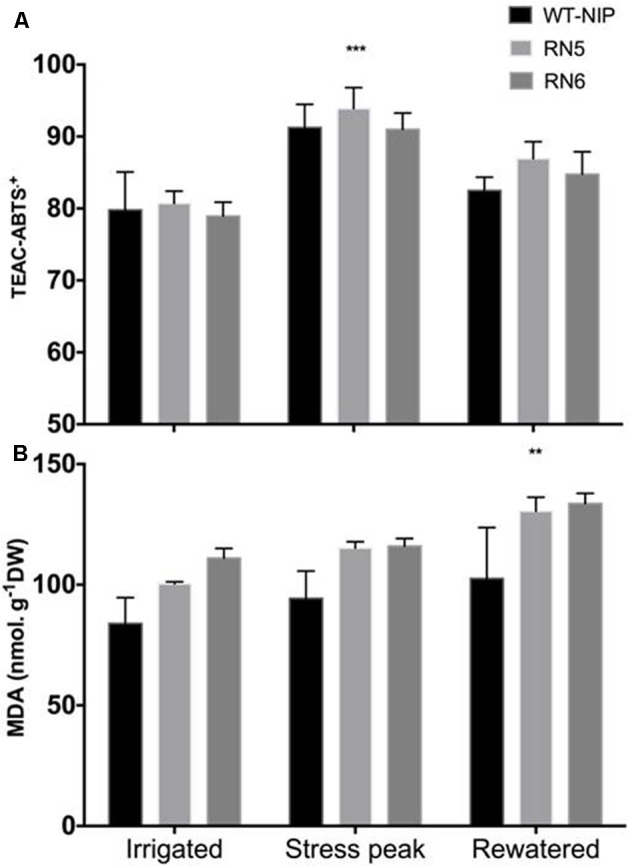
Antioxidant capacity determined by radical cation ABTS^∙+^ and MDA content under irrigated, drought, and rewatered conditions in Trial 2. **(A)** Drought stress induced a significant increase (*p* < 0.001) in the antioxidant capacity, measured by the ABTS assay. No significant differences were detected among lines. **(B)** A significant increase in MDA (*p* < 0.01) was measured in all lines after rewatering. Significance level ^∗∗^*p* < 0.01, ^∗∗∗^*p* < 0.001.

Based on these results, in Trial 3 we evaluated whether a superior capacity for removal of reactive-oxygen-species (ROS) was involved in the enhanced drought tolerance observed in AlSAP-lines. We quantified the content of MDA in leaves to monitor the degree of oxidative stress induced by drought (**Figure 7**). Leaf MDA concentrations increased in response to drought in all lines and continued to rise after rehydration (*p* < 0.01). RN5 and RN6 exhibited significantly higher MDA content than NT controls before the drought, at the peak of drought-stress, and 10 days after rewatering (*p* < 0.01). No association was observed between the concentration of MDA and chlorophyll content (data not shown). All plants exhibited decreases in chlorophyll content during the experiment; however, no significant differences were observed among lines (data not shown).

We also evaluated the capacity of plants to remove ROS using the ABTS^•+^-based system, which estimates total antioxidant capacity (Tirzitis and Bartosz, 2010). No significant differences were observed among lines at any time point (**Figure 7**); however, a marked increase in antioxidants was observed in response to drought. The antioxidant capacity decreased after rewatering, although it remained higher than that observed before stress, possibly due to stress priming (Wang et al., 2014).

## Discussion

### GY Is Protected from Field Drought at the Reproductive Stage by AlSAP

In this investigation, we characterized the field performance of rice lines (*O. sativa* L. ‘Nipponbare’) transformed with the *AlSAP* gene isolated from the halophyte grass *A. littoralis*. Our results demonstrate that the constitutive expression of *AlSAP* did not involve growth, development or phenology trade-offs, and confirm a role for AlSAP in enhancing rice tolerance to drought stress.

*AlSAP* expression improved the tolerance of rice to drought stress imposed at the reproductive stage, and this effect translated into higher GY. In our three independent field trials, AlSAP-lines achieved higher GYs than NT and WT controls when exposed to drought-stress of different intensities, complementing previous findings by Ben Saad et al. (2012) indicating superior drought tolerance at the vegetative stage. The higher GY corresponded with a significant increase in panicle number and spikelet fertility. No differences were observed in the number of spikelets per panicle, nor in individual grain weight. We disregarded the hypothesis that differences in panicle number and spikelet fertility were due to phenology, as all the lines flowered at around the same time and drought stress began at heading, when the number of panicles was already determined. In fact, we observed no difference in panicle number between plants grown under irrigated and drought conditions for either WT-NIP or AlSAP-lines.

For RN2, RN4, and RN5, the same ranking in stress tolerance (RN5 > RN2 > RN4) correlating with levels of *AlSAP* transcript accumulation was observed under field drought conditions and previous laboratory and greenhouse experiments (Ben Saad et al., 2012). The RN6 line, harboring the *AlSAP* coding sequence under the control of the rice *ACT1* promoter, performed similarly to RN5. In contrast, somaclonal variation in *Ubi1* NT controls, led to inferior performance under drought conditions. This depressed background likely prevented expression of the full benefit of a low level of AlSAP accumulation in the transgenic lines. Variation among plants derived from tissue culture is an extensively reported phenomenon, including in transgenic lines tested for drought-tolerance (Xiao et al., 2007, 2009), and has both genetic (Miyao et al., 2012) and epigenetic (Stroud et al., 2013) origins. These reports stress the requirement for evaluation of homozygous transgenic lines alongside their azygous, sibling progenies.

### Drought Protection in AlSAP-Lines Cannot Be Explained by Maintenance of Photosynthesis

Photosynthesis and yield are positively correlated in rice under both irrigated and drought conditions (Centritto et al., 2009; Gu et al., 2012). However, the GY differences observed between AlSAP-lines and WT-NIP under drought-stress were not clearly associated with *A*_sat_ in flag leaves. This result contrasts with those obtained by Ben Saad et al. (2012), which demonstrated enhanced photosynthetic performance in RN5 under mild and severe drought applied at the vegetative stage, a response engendered by the capacity of this transgenic line to maintain elevated *G*_s_. Similar to that report, we found that stomatal closure limited *A*_sat_ under drought conditions; however, we did not detect an improved capacity to maintain open stomata in AlSAP-lines. Furthermore, evaluation of *A*_max_ during drought and after rehydration indicated that similar non-stomatal limitations operate in AlSAP-lines and WT-NIP under severe stress conditions.

Although we did not observe differences in instantaneous *A*_sat_ on a single leaf basis, it is still possible that AlSAP-lines may have achieved improved photosynthetic performance. RN5 and RN6 exhibited moderate LR, which can increase photosynthesis and GY in rice (Zhang et al., 2009; Zou et al., 2011). LD was also lower in AlSAP-plants, and they were able to recover their vegetative biomass almost entirely after drought. Additionally, they showed significantly higher above-ground dry matter accumulation under both irrigated and drought conditions, indicating that increased biomass contributed to carbon assimilation and GY. This combination of traits may have led to higher carbon fixation rates, at either the leaf or the whole plant basis, in AlSAP-lines, particularly during the active grain filling phase, which occurred close to the peak of drought-stress.

Delayed LR indicates an ability to sustain turgor, despite drought-stress; for example, through increased water uptake or osmotic adjustment. We did not observe differences in Ψw that could explain the reduction of LR in AlSAP-lines, although Ψw was slightly higher in RN6 at stress peak. However, it is interesting that AlSAP-lines maintained Ψw values similar to those of WT-NIP, despite their elevated biomass accumulation, which would be expected to reduce this characteristic (Pantuwan et al., 2002). Neither was differential regulation of *G*_s_ observed in AlSAP-lines; hence, the reduced LR may be associated with improved access to water in the soil. The characterization of the response of AlSAP-seedlings to different abiotic stresses demonstrated their improved ability to maintain root growth and development (Ben Saad et al., 2012). Thus, it would be interesting to evaluate the development of the root system of these plants in the field.

It is important to highlight that *AlSAP* expression reduced LR but did not affect leaf morphology or plant development (data not shown; Supplementary Figure S2). A similar effect has been reported in rice plants overexpressing the *Arabidopsis* TFs CBF3/DREB1A (CBF3), ABF3, and SDIR1 (Oh et al., 2005; Zhang et al., 2009), or OsNAC TFs (Hu et al., 2006; Jeong et al., 2010). The molecular mechanisms underlying this effect remain unknown, but may include the interaction of some of these, or similar TFs, with other components in drought-stress signaling pathways, in which SAPs may also participate.

### Developmental Stage and Environmental Conditions May Influence the Protective Effect of AlSAP on Photosynthesis

The imposition of stress at different phenological stages may influence plant responses to drought and the mechanisms by which AlSAP contributes to drought tolerance. Ben Saad et al. (2012) performed physiological investigations on the last expanded leaf of AlSAP rice plants at the 5–6 leaf, early tillering stage. In contrast, we evaluated photosynthesis parameters using the flag leaf, which is physiologically different to other leaves, and suffers distinctly under stress conditions. Hormone balance, oxidative damage, water regulation, and particularly drought-induced changes in photosynthesis, proceed distinctly in the flag leaf (Chaves et al., 2003; Biswal and Kohli, 2013). Drought-stress during anthesis reduces the rate of photosynthesis of the flag leaf and increases the remobilization of assimilates from it to sustain grain demand, accelerating its senescence, which has further impacts on photosynthesis (Peng and Ismail, 2004). In contrast, during vegetative growth and especially in young leaves, drought depresses the photosynthesis rate without inducing senescence (Peng and Ismail, 2004). The mechanisms involved in reducing photosynthesis are different from those that enhance remobilization, and do not necessarily involve AlSAP.

Evaluation at distinct physiological stages may also have involved developmentally associated differences in *AlSAP* transcription levels or expression patterns. We found that a higher accumulation of transcript correlates positively with drought-tolerance in AlSAP-lines. Similar observations were reported for rice, tobacco, and durum wheat (Ben Saad et al., 2010, 2011, 2012). These results may indicate that complete expression of the effects of AlSAP depends to some degree on gene transcript, or protein, accumulation. Alternatively, expression in certain organs/tissues or at specific growth stages may be required for AlSAP to achieve its full potential.

Co-occurrence of multiple abiotic stresses is another factor to consider. In the field, crops encounter a combination of stresses that could require different or antagonistic responses, and consequently may activate varying molecular mechanisms (Mittler, 2006; Mazzucotelli et al., 2008; Salekdeh et al., 2009). Drought and heat stresses are classic examples of this kind of interaction that may elicit antagonistic physiological responses and activate the transcription machinery in a non-additive fashion (Atkinson and Urwin, 2012). In our field trials, plants coped with both drought and high diurnal temperatures. Although, AlSAP appeared to confer tolerance to both heat and drought stresses applied independently to transformed tobacco (Ben Saad et al., 2010), its effect in response to the simultaneous imposition of these factors has not been tested and cannot be predicted from these individual studies (Salekdeh et al., 2009).

It is also possible that the improved yields observed in AlSAP-lines exposed to drought during the reproductive stage is not the result of better performance at the time of stress, but rather signifies a primed status, acquired during vegetative growth, due to the higher accumulation of carbon reserves resulting from enhanced photosynthetic performance at the vegetative stage.

### AlSAP as a Candidate for Reduction of Yield Loss under Drought-Stress

Evidence gathered to date demonstrates that *AlSAP* overexpression in rice is effective in conferring drought tolerance at both the vegetative and reproductive stages. Stress protection during distinct phenological stages was also reported for *AlSAP*-transformed tobacco and durum wheat. Importantly, *AlSAP* expression exerted similar effects on growth and yield of durum wheat, tobacco, and rice, under both normal and stressed conditions (Ben Saad et al., 2010, 2011, 2012). These similar responses indicate that the beneficial role of AlSAP in regulating growth and development may be universal in monocotyledonous and dicotyledonous plants.

The gains in GY in *AlSAP*-transformed rice ranged from 26 to 115%, depending on the line and the severity of the stress exerted in the field. These gains are very high compared with reports for other transgenic rice lines expressing genes conferring drought tolerance and challenged with milder stress intensity, as indicated by the GY loss observed in controls under drought vs. standard conditions (see **Table 1** for AlSAP-lines). For example, overexpression of *AP37* in rice enhanced drought tolerance at the reproductive stage and resulted in yield gains of 16–57% above controls. However, in this study, the stress intensity was limited, since plants remained irrigated to avoid LR, resulting in a GY loss of only 32% in the WT line (Oh et al., 2009). In another study, rice plants overexpressing *OsNAC10* showed enhanced drought tolerance at the reproductive stage, and GY increased by 25–42%; however, again milder stress conditions were applied (Jeong et al., 2010). The expression of *HYR*, which afforded a 14–39% GY increase under stress, was also evaluated under mild drought stress, since GY in the control line reduced by only 30% (Ambavaram et al., 2014).

A limited number of studies applied drought intensity more comparable to our experimental conditions. Plants overexpressing *LOS5* and *ZAT10* showed gains ranging from 11 to 36% compared with their controls, which suffered an 82% yield reduction (Xiao et al., 2009). Plants overexpressing *AtEDT1/HDG11* exhibited a GY gain of 65% over the control, the yield of which was reduced by 83% when subjected to severe drought (Yu et al., 2013). More remarkably, rice lines expressing *OsCPI1* showed a 2.5–3-fold superior GY over the control, the yield of which dropped by 90% under severe drought conditions (Huang et al., 2007).

Grain production penalties, due to panicle sterility and abnormalities, under unstressed conditions have frequently been reported when transgenic TFs are constitutively expressed (Dubouzet et al., 2003; Oh et al., 2009; Park et al., 2013). In our study, the use of three distinct promoters, *CaMV35S*, maize *Ubi-1*, and rice *ACT1*, inducing a range of constitutive gene expression levels, imposed no penalties in either rice growth or GY under irrigated conditions. Conversely, we observed modest, but consistently higher GY in *AlSAP* rice lines under paddy fields. These results contrast with the effects reported previously from several studies of SAP over-expression. To date, ectopic expression of five (*OsSAP1, OsSAP8, OsSAP7, OsSAP9*, and *OsSAP11*) of the 18 rice *SAP* genes has been investigated in heterologous or homologous systems. Two examples of SAPs (*OsSAP1* and *OsSAP8*) over-expressed in rice under the maize ubiquitin promoter resulted in an enhanced tolerance to abiotic stresses, but detrimental effects on GY under standard growth conditions (Kanneganti and Gupta, 2008; Dansana et al., 2014). A third example (*OsSAP7*) demonstrated a negative effect of SAP overexpression in *Arabidopsis* abiotic stress tolerance, due to its role as negative regulator of ABA responses (Sharma et al., 2015).

The fact that these detrimental effects are absent in rice plants expressing the *AlSAP* gene underlines the potential for the application of AlSAP transgenic lines to reduce yield losses in crop species facing environmental stress.

### Conclusion and Prospects

This is the first report of field evaluation of transgenic plants expressing an A20/AN1 stress-associated protein. We consistently observed that AlSAP accumulation enhanced GY under drought, and did not penalize either yield or plant growth under irrigated conditions. Improved GY was associated with higher biomass accumulation, a greater number of productive panicles, and lower panicle sterility, even under severe stress. Milder environments should be tested since it is possible that further GY gains can be attained by *AlSAP* expression under more favorable conditions. It will also be important to introduce *AlSAP* in other genetic backgrounds, notably elite upland cultivars with established basal levels of drought-tolerance, to determine whether AlSAP can further enhance their ability to resist drought.

Recent reports of transgenic protection of rice from drought-stress associated beneficial transgene effects with stronger root systems (Jeong et al., 2010, 2013; Redillas et al., 2012; Ambavaram et al., 2014). The influence of AlSAP on root development and hydraulic conductance require further assessment in specifically designed experiments. Also, a combination of transgenes with different modes of action, and that improve distinct traits contributing to yield under drought could be attempted.

We note apparent discrepancies in the effects of *AlSAP* in protection of photosynthesis observed in our greenhouse and field experiments. Several hypotheses to explain these findings have been presented and require further exploration. To provide additional clues regarding the physiological and molecular mechanisms operating in AlSAP crops, a better understanding of *AlSAP* gene function is required; in particular, investigations to identify proteins interacting with AlSAP in rice and decipher its regulation network will be beneficial. Such studies will provide opportunities to consolidate the protection afforded by AlSAP through stress-inducible/developmentally regulated expression strategies and combined expression with other genes (e.g., NAC genes) affording abiotic stress tolerance through different modes of action.

## Author Contributions

TG-H and EG conceptualized and wrote the manuscript. TG-H, AP, SO, MR, and DM performed the experiments. MS, DF, WB, RB, MI, JT, AAD, and AH, revised the paper critically. All authors revised and approved the final manuscript.

## Conflict of Interest Statement

The authors declare that the research was conducted in the absence of any commercial or financial relationships that could be construed as a potential conflict of interest.

## Footnotes

1 http://faostat3.fao.org/

2 http://ricephenonetwork.irri.org/
