# Supplementary material for: Expression of the Aeluropus littoralis AlSAP Gene Enhances Rice Yield under Field Drought at the Reproductive Stage
Source: Front Plant Sci. 2017 Jun 12;8:994. doi: 10.3389/fpls.2017.00994 (PMC5466986; doi:10.3389/fpls.2017.00994)
Supplement: Supplementary file 1 [file Table_1.DOCX]

**Table S1** Primers used in RT-PCR and Q-PCR analyses. The Accession no. of the housekeeping gene, *OsExp*, is Os06g11070*.*

| ***RT-PCR*** | ***Q-PCR*** |
| --- | --- |
| ***AlSAP-5’F****: GGAAAAGTCGCGTGCCGT* | ***QAlSAP-F*** *: CCTGCACCGTTATACGGACT* |
| ***AlSAP-3’ R****: GACCCAGCCCGTGAAGGG* | ***QAlSAP-R*** *: TCTGCTTGGCAATCTGCTCC* |
|  | ***OsEXP-F*** *: AGGAACATGGAGAAGAACAAGG* |
|  | ***OsEXP-F*** *: CAGAGGTGGTGCAGATGAAA* |
