# Supplementary material for: Expression of the Aeluropus littoralis AlSAP Gene Enhances Rice Yield under Field Drought at the Reproductive Stage
Source: Front Plant Sci. 2017 Jun 12;8:994. doi: 10.3389/fpls.2017.00994 (PMC5466986; doi:10.3389/fpls.2017.00994)
Supplement: Supplementary file 2 [file Table_2.DOCX]

| *Trial 2-Rainout shelter-CIAT* | | | | | |
| --- | --- | --- | --- | --- | --- |
| **Source of variation** | **DF** | **SS** | **MS** | **F** | **P** |
| *Days after last irrigation (DAI)* | 6 | 31.40 | 5.23 | 16.68 | 0.000 |
| *Repetition (R)* | 2 | 1.15 | 0.57 | 1.83 | 0.173 |
| *Interaction* | 12 | 3.79 | 0.32 | 1.01 | 0.459 |
| *Error* | 42 | 13.18 | 0.31 |  |  |
| *Total* | 62 | 49.52 |  |  |  |
|  | | | | | |
| *Trial 3-Rainout shelter-CIAT* | | | | | |
| **Source of variation** | **DF** | **SS** | **MS** | **F** | **P** |
| *Days after last irrigation (DAI)* | 5 | 223.03 | 44.61 | 6.32 | 0.000 |
| *Repetition (R)* | 5 | 75.69 | 15.14 | 2.15 | 0.070 |
| *Interaction* | 25 | 160.38 | 6.41 | 0.91 | 0.592 |
| *Error* | 72 | 507.98 | 7.05 |  |  |
| *Total* | 107 | 967.081 |  |  |  |

**Table S2** ANOVA of the effects of drought treatment (days after the last irrigation) and repetition over soil water potential (SWP) measured at 40 cm depth in Trials 2 and 3. Changes in SWP for these trials are presented in Figure 1.
