# Supplementary figures and images for: Expression of the Aeluropus littoralis AlSAP Gene Enhances Rice Yield under Field Drought at the Reproductive Stage
Source: Front Plant Sci. 2017 Jun 12;8:994. doi: 10.3389/fpls.2017.00994 (PMC5466986; doi:10.3389/fpls.2017.00994)

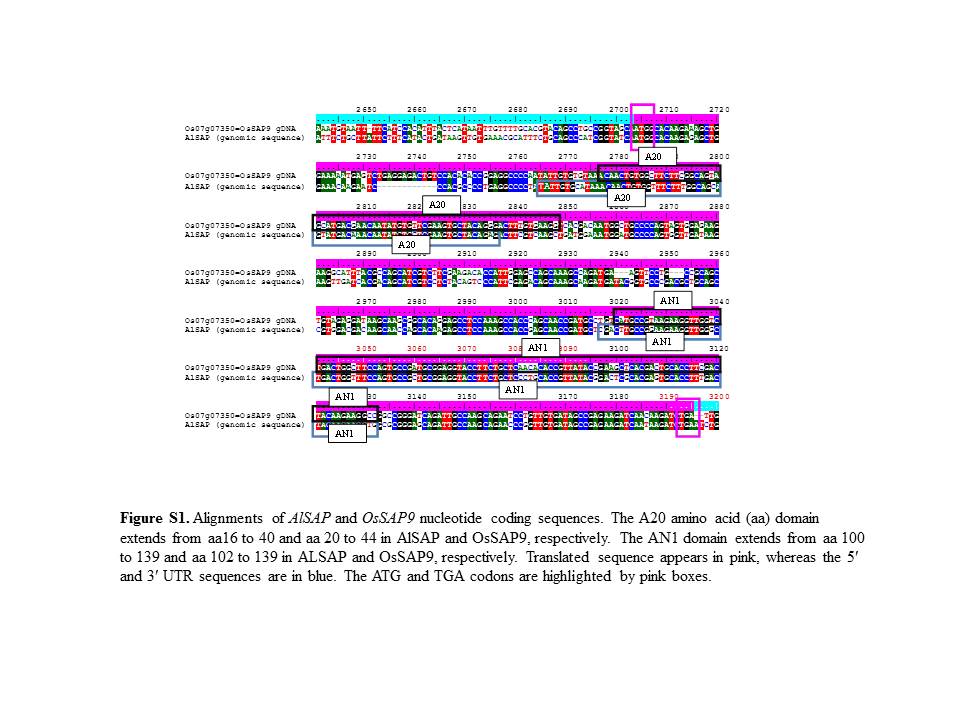

Supplement: Supplementary file 3 [file Image_1.JPEG]

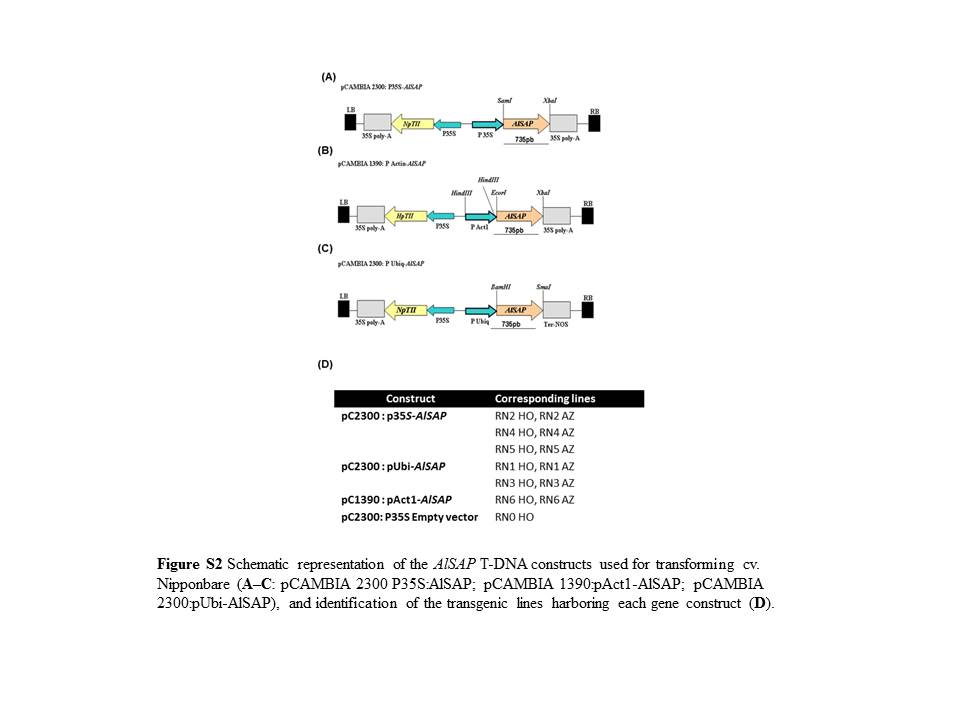

Supplement: Supplementary file 4 [file Image_2.JPEG]

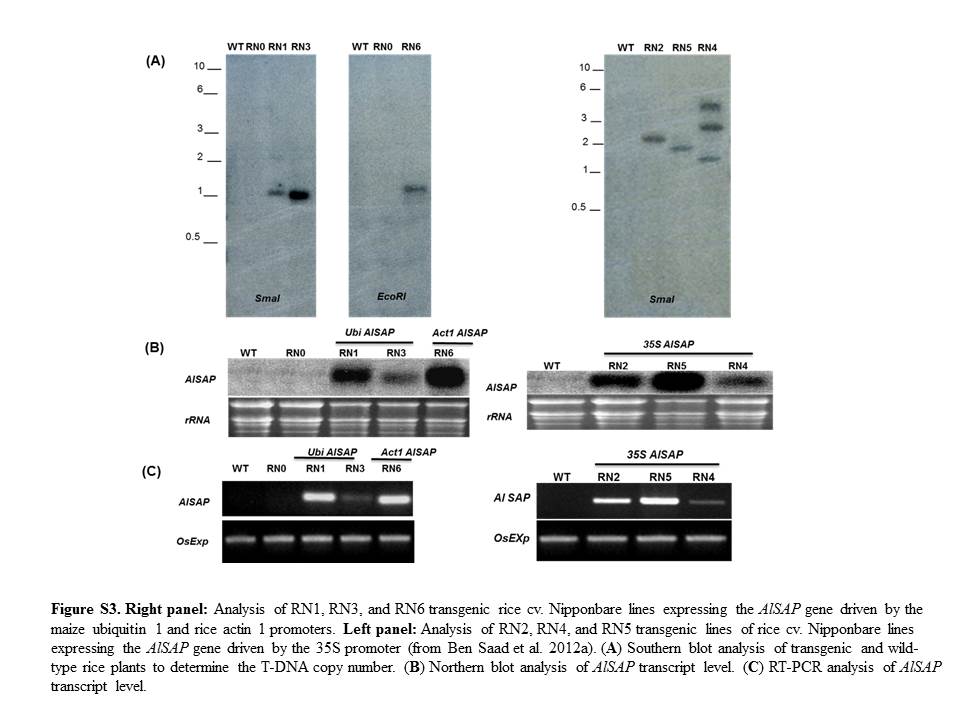

Supplement: Supplementary file 5 [file Image_3.JPEG]

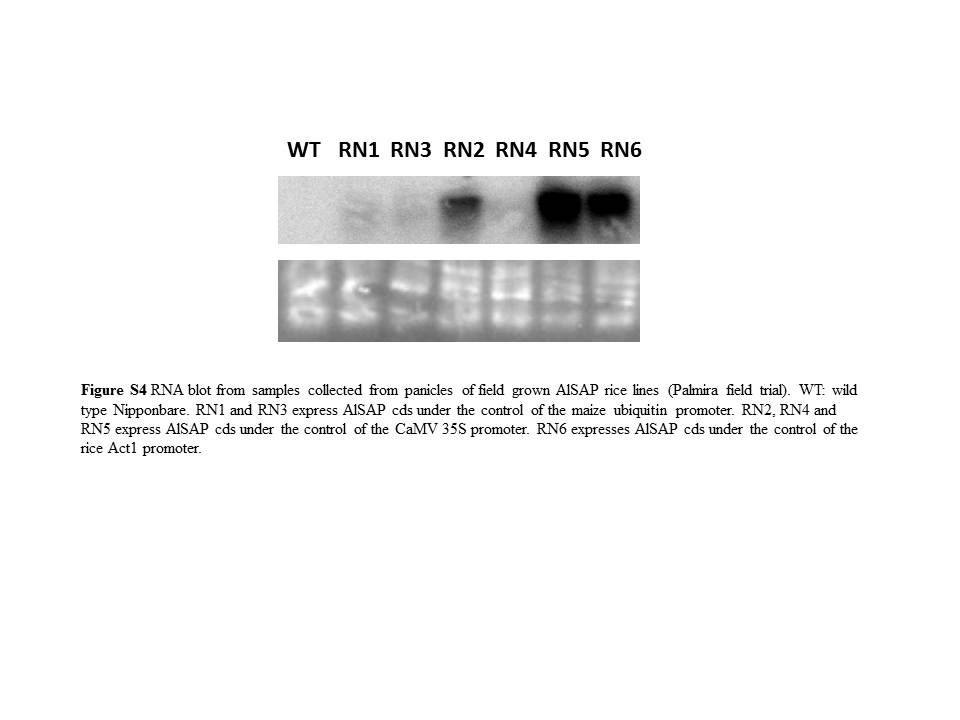

Supplement: Supplementary file 6 [file Image_4.JPEG]

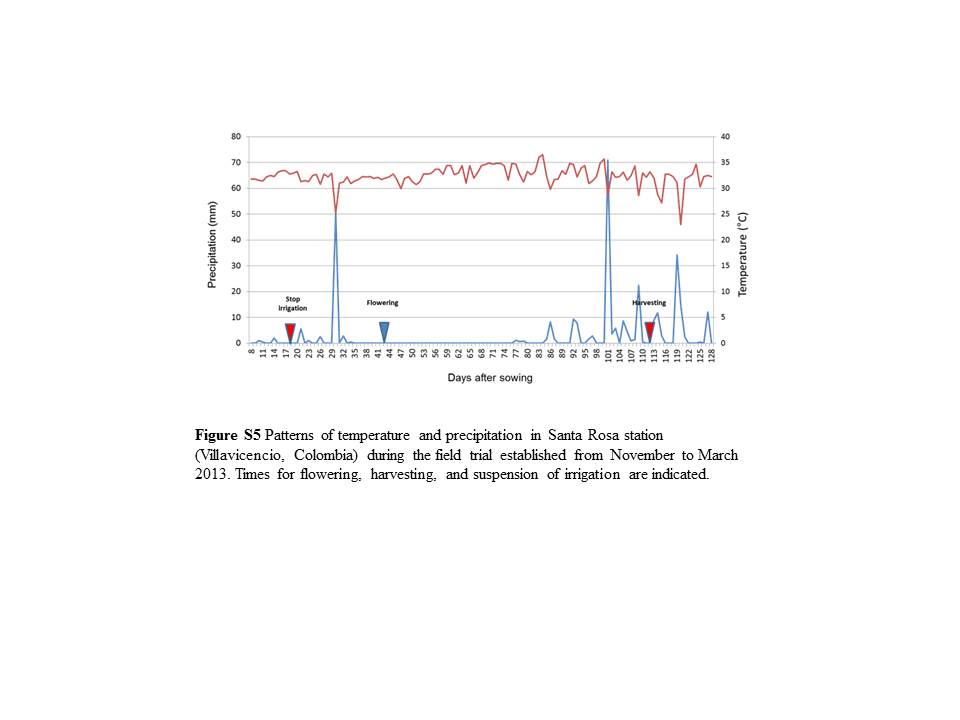

Supplement: Supplementary file 7 [file Image_5.JPEG]

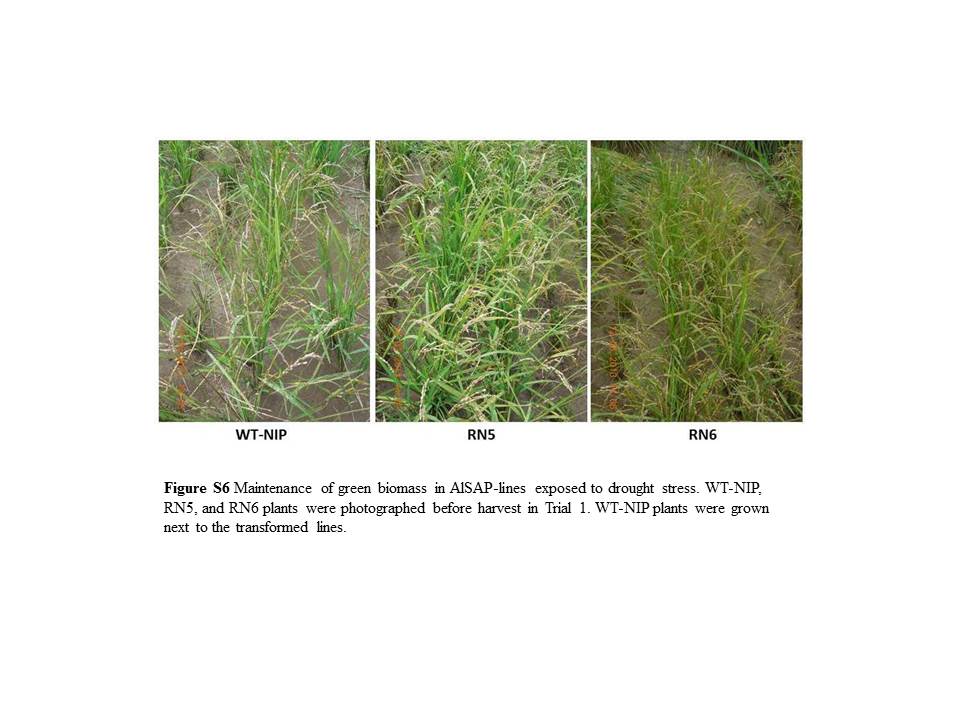

Supplement: Supplementary file 8 [file Image_6.JPEG]
